# DockTope: a Web-based tool for automated pMHC-I modelling

**DOI:** 10.1038/srep18413

**Published:** 2015-12-17

**Authors:** Maurício Menegatti Rigo, Dinler Amaral Antunes, Martiela Vaz de Freitas, Marcus Fabiano de Almeida Mendes, Lindolfo Meira, Marialva Sinigaglia, Gustavo Fioravanti Vieira

**Affiliations:** 1 Núcleo de Bioinformática do Laboratório de Imunogenética, Departamento de Genética, Universidade Federal do Rio Grande do Sul, Postcode 91501-970, Brazil; 2 Department of Computer Science, Rice University, Houston, Texas, 77005, USA; 3 CESUP-Centro Nacional de Supercomputação, Universidade Federal do Rio Grande do Sul, Postcode 90035-190, Brazil

## Abstract

The immune system is constantly challenged, being required to protect the organism against a wide variety of infectious pathogens and, at the same time, to avoid autoimmune disorders. One of the most important molecules involved in these events is the Major Histocompatibility Complex class I (MHC-I), responsible for binding and presenting small peptides from the intracellular environment to CD8^+^ T cells. The study of peptide:MHC-I (pMHC-I) molecules at a structural level is crucial to understand the molecular mechanisms underlying immunologic responses. Unfortunately, there are few pMHC-I structures in the Protein Data Bank (PDB) (especially considering the total number of complexes that could be formed combining different peptides), and pMHC-I modelling tools are scarce. Here, we present DockTope, a free and reliable web-based tool for pMHC-I modelling, based on crystal structures from the PDB. DockTope is fully automated and allows any researcher to construct a pMHC-I complex in an efficient way. We have reproduced a dataset of 135 non-redundant pMHC-I structures from the PDB (Cα RMSD below 1 Å). Modelling of pMHC-I complexes is remarkably important, contributing to the knowledge of important events such as cross-reactivity, autoimmunity, cancer therapy, transplantation and rational vaccine design.

The immune system is mainly responsible for defending the organism against a wide range of infectious pathogens, such as viruses, bacteria and fungi. At the same time, it should be able to preserve the organism, avoiding autoimmunity events, for example. This complex system is orchestrated by a set of cells and molecules involved in clearing infections and maintaining a healthy organism. One of these molecules with pivotal importance is the Major Histocompatibility Complex class I (MHC-I), which is typically capable to bind short peptides with eight to twelve amino acids in length (also called epitopes in this context). The peptide:MHC-I (pMHC-I) complex is transported through a specific endogenous pathway to the cell surface, where it can be inspected by a T Cell Receptor (TCR) of a CD8^ + ^lymphocyte^1^. Based on complementary structural patterns, the pMHC-I and TCR interaction can trigger an immunologic response, which will mainly depend on the peptide source^2^,^3^,^4^.

The MHC-I molecule is composed of an α domain (subdivided in α1, α2 and α3 regions), encoded by one of the most polymorphic regions of the genome, referred to as MHC locus, located on chromosome 6 (in humans) and chromosome 17 (in murines)^5^,^6^. Additionally, a β2-microglobulin interacts with the MHC-I α domain providing complex stability^7^. The protein’s polymorphism occur mainly in the α1 and α2 regions, which form a cleft where the peptide is bound and presented to the TCR. Each MHC-I allele encodes a specific protein, called allotype. The MHC-I allotypes are highly variable in terms of amino acids composition and, depending on the organism studied, a specific name is assigned, such as Human Leukocyte Antigen (HLA), in humans, and H-2 antigen (H-2), in murines.

The MHC-I allotype variability allows a broad array of peptides to bind inside the MHC-I cleft through a specific interaction pattern between the epitope and the MHC-I residues. Thus, the understanding of the epitope binding mode and the structural features of this protein complex is of pivotal importance to unveil the molecular basis underlying important immune responses. Unfortunately, the low number of pMHC-I structures experimentally resolved and the lack of accessible and reliable structural *in silico* modelling approaches are hindering for the evolution of this field. Currently, three-dimensional structures of pMHC-I complexes are determined through specific techniques, such as X‐ray crystallography and nuclear magnetic resonance (NMR) spectroscopy, which are costly and time-consuming. To overcome this, pMHC-I modelling represents an interesting and creative solution. Despite the availability of homology modelling techniques, each allotype presents specific particularities that cannot be simply determined through regular approaches. Thus, molecular modelling requires a careful structural study based on a solid validation process, which should take the wide MHC-I allotype variability in consideration. Immunoinformatics programs devoted to pMHC-I modelling have been developed or are under development^8^,^9^,^10^,^11^,^12^,^13^,^14^,^15^,^16^,^17^, but there is currently few online programs available to the scientific community.

Here, we present DockTope, a fully automated web-server tool designed with the purpose of modelling pMHC-I complexes for two human (HLA-A*02:01 and HLA-B*27:05) and two murine (H-2-Db and H-2-Kb) MHC-I allotypes. We have validated this tool through the cross-docking reproduction of 135 non-redundant structurally resolved pMHC-I structures available in the Protein Data Bank (PDB), using Cα and all atom Root Mean Square Deviation (RMSD) values for evaluation. DockTope has been fully automated using different programming languages, such as python and shell scripting. In addition, we have designed a dedicated web server providing free and easy access to any user throughout the world.

## Results

### The rationale behind DockTope

**Figure 1:**
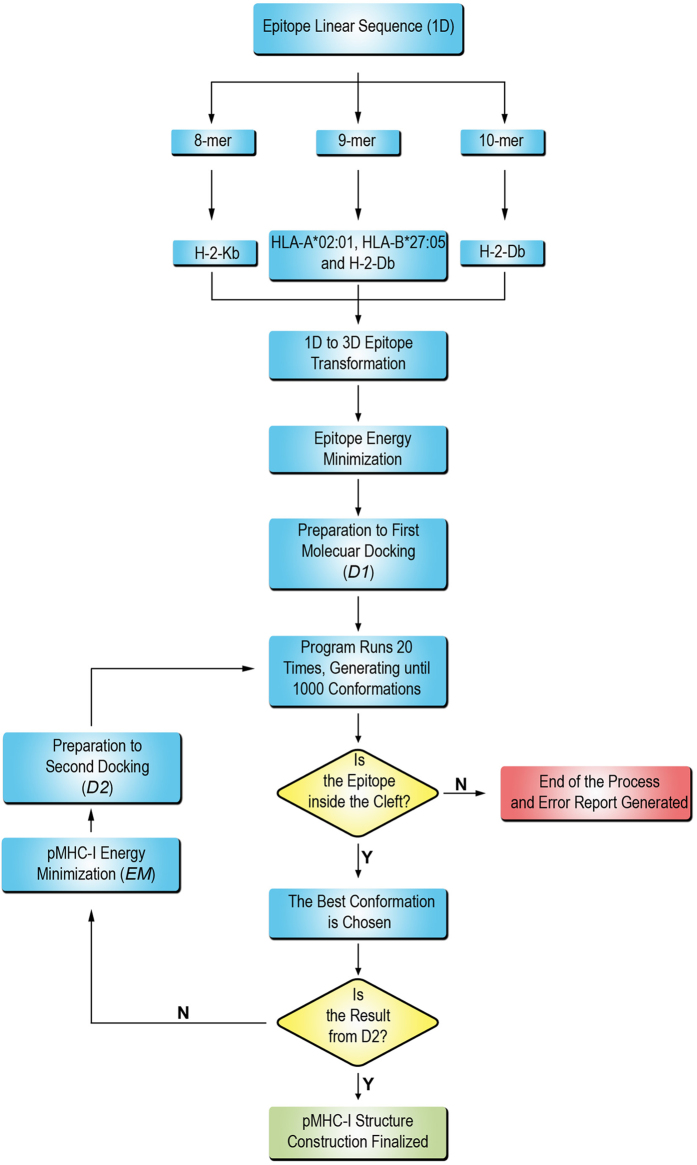
Flowchart showing the DockTope sequence of steps. The program starts from the linear sequence of the epitope and terminates when the pMHC-I structure is obtained. The user chooses among the allotypes HLA-A*02:01, HLA-B*27:05, H-2-Db and H-2-Kb, which depends on the epitope length (8-mer, 9-mer or 10-mer). The program prepares the files for the first docking (D1), where the best suited conformations will be saved during 20 rounds of simulation. The program checks whether the epitope is inside the cleft. In case of error, a report is written and the program stops. Otherwise, the program proceeds to the next step, where the best conformation is chosen. The program checks whether the structure generated is coming from D1. If so, the structure is energy minimized (EM) and a second docking is performed. In the end, the pMHC-I structure is generated in the PDB format.

The DockTope tool is based on the D1-EM-D2 approach^18^, a pMHC-I modelling technique published by our group in 2010. The D1-EM-D2 approach is based on a protocol that employs a molecular docking step (D1), followed by an energy minimization (EM) of the pMHC-I complex and a final molecular docking round (D2). To develop DockTope, the D1-EM-D2 approach has gone through several implementations, including a new data validation improving its accuracy and reliability, and the full automation of the process. Here, we briefly describe important highlights of the technique improvement. A flowchart of the whole process is provided in Fig. 1.

Before the D1 step, the user provides the linear epitope sequence to be modelled, which is transformed into a three-dimensional structure. This is made possible by the fact that each epitope carries a specific backbone structure, depending on the MHC-I allotype where it is presented^18^. The epitope to be modelled is superimposed on another epitope (named here the ‘Epitope pattern’), which was already determined by X‐ray crystallography in the context of the target MHC-I (Table 1). Since the three-dimensional epitope structure is obtained by modelling the side chains over a constrained backbone, a brief energy minimization step is performed, allowing for a mild global relaxation of the peptide.

To perform the D1 step, all the MHC-I residues and the epitope backbone are kept rigid. Only the epitope side chains are allowed to move. During the molecular docking, the epitope can also perform rotational and translational movements, and the program AutoDock Vina^19^ is used to search for the best epitope conformations inside the MHC-I cleft region. One round of molecular docking provides the best epitope conformations based on a scoring function, returning a Binding Energy (BE) value in kcal/mol, which is used by the program to rank the best conformations. To improve chances to find a suitable conformation, our approach performs twenty independent docking runs, using different initial points, which ensures that the program will search a wider range of conformations. In the end, the best conformation of each docking run is retrieved, producing a total of twenty structures.

Before the pMHC-I EM, the best epitope conformation is chosen among the twenty structures generated by AutoDock Vina. This choice is based on two variables: the BE and the average RMSD of each conformation in relation to all other structures. Since the epitope conformation having the best interaction with the MHC-I presents a low BE (a basic relationship between entropy and free energy calculations of non-covalent binding^20^), we have designed a specific shell script to calculate the BE average among the twenty epitope structures generated from D1. This way we obtain a cut-off (*Co*) value that is used as a first structure filter. This will exclude spurious conformations that can arise and that do not represent the binding mode of the epitope with its respective MHC-I. Additionally, the remaining epitope conformations are compared with each other, using the *g_confrms* program from the GROMACS package^21^, which returns a RMSD value for each conformation pair. The epitope conformation with the lowest RMSD mean among all outputted conformations (i.e. the average structure) is chosen as the best structure. We describe the equation for choosing the best conformation in the ‘Material and Methods section’. After that, the pMHC-I complex is submitted to an EM protocol, where all the residues are kept flexible to accommodate and correct the interactions between epitope and MHC-I.

The second and final molecular docking round, D2, is performed in the same way as the D1. The D2 step is intended to refine the pMHC-I structure, which is possible because the EM has already accommodated the MHC-I side chains to the target epitope structure. The final structure with the best epitope conformation is also chosen as explained above.

### Automation and validation of DockTope

Back in 2010, the D1-EM-D2 approach validation process was performed over 46 pMHC-I structures available in the PDB, including the HLA-A*02:01, H-2-Db and H-2-Kb allotypes^18^. Here, thanks to the DockTope automated process, a broader validation analysis is reported over 135 pMHC-I structures, encompassing the previous dataset and including the HLA-B*27:05 allotype (Table 2). This represents almost three times the number of structures previously analysed. Also, the automation process first presented here, is a crucial feature of the DockTope web-based tool; it was made possible by writing and concatenating of more than 20 shell and python scripts. After submitting a sequence, the program automatically generates the three-dimensional epitope structure, according to the MHC-I of interest, and performs the molecular docking and energy minimization steps.

Since our method uses a reference MHC-I structure to build every model of a given allotype (also referenced here as ‘MHC donor’), the validation process occurred through a cross-docking scheme. Each pMHC-I structure was modelled using only the epitope linear sequence as input and, in the end, the generated pMHC-I complex (model) was compared to its respective crystal structure deposited in the PDB (target). The comparison was based on RMSD values for the epitope atoms (considering Cα or all atoms) following the MHC-I chains superposition of model and target. This way, it was possible to obtain a RMSD value taking into consideration not only conformational changes on the modelled epitope, but also translational and rotational differences inside the MHC-I cleft.

**Figure 2:**
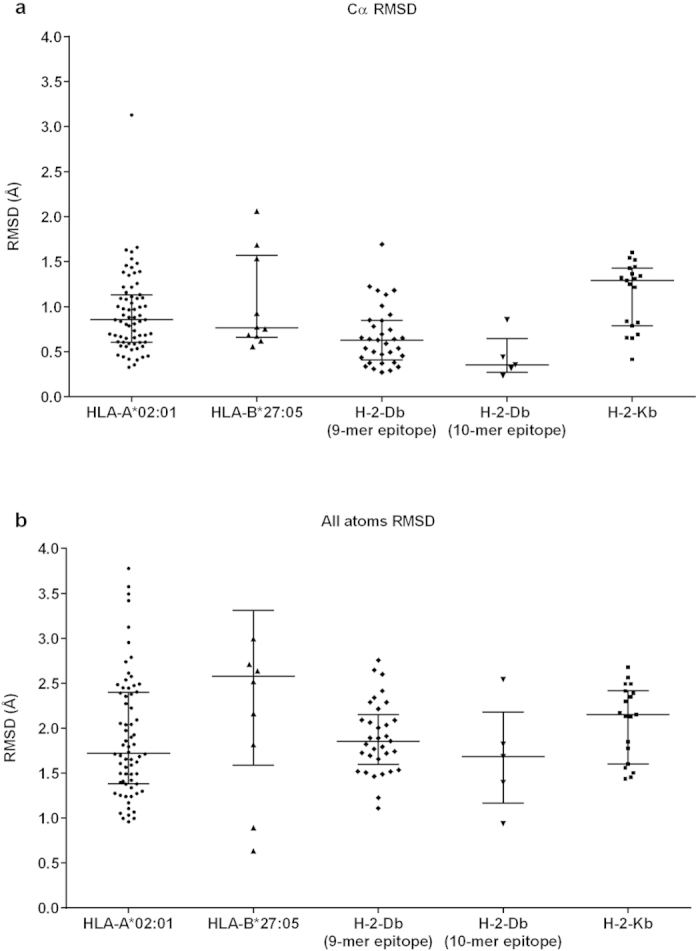
Scatter dot plot representing the DockTope validation values for 135 pMHC-I structures from the PDB. The validation process was performed through cross-docking, considering the Cα (**a**) and all atoms (**b**) RMSD for each epitope. Each point represents the value for a reproduced structure. The statistic data are shown as a median with interquartile range (25% to 75%). On the y-axis, RMSD stands for Root Mean Square Deviation; on the x-axis, the MHC types are represented.

A cut-off of 2 Å or less was used to indicate the accuracy of the modelling approach. As observed in Table 1, the reproduction of all MHC-I allotypes produced average Cα RMSD values below 2 Å. There were only two outliers, with Cα RMSD values of 3.129 Å and 2.061 Å, respectively corresponding to the attempts of reproducing an HLA-A*02:01 (PDB ID: 2GTW) and an HLA-B*27:05 (PDB ID: 3BP4) peptide-loaded complex. When all atoms were considered, the RMSD values were slightly higher in comparison to Cα RMSD values. This was expected since epitope side chains could present high flexibility in the MHC-I cleft, especially the residues involved in the interaction with the T Cell Receptor (TCR)^22^. Considering all modelled epitopes, the overall RMSD average was 0.882 Å ± 0.437 Å (s.d.) and 1.964 Å ± 0.655 Å (s.d.) for Cα and all atoms, respectively. As observed in Fig. 2, which shows the data distribution around the median with interquartile range, most of the Cα RMSD measurements were grouped even below 1.5 Å, which strongly highlights the precision of the DockTope modelling approach. Of note, the median for Cα/all atoms RMSD values were 0.854 Å/1.723 Å, 0.764 Å/2.578 Å, 0.629 Å/1.856 Å, 0.355 Å/1.685 Å and 1.292 Å/2.153 Å for HLA-A*02:01, HLA-B*27:05, H-2-Db (9-mer epitope), H-2-Db (10-mer epitope) and H-2-Kb, respectively. All 135 RMSD values for the evaluated structures are available in Supplementary Table S1 online.

**Figure 3:**
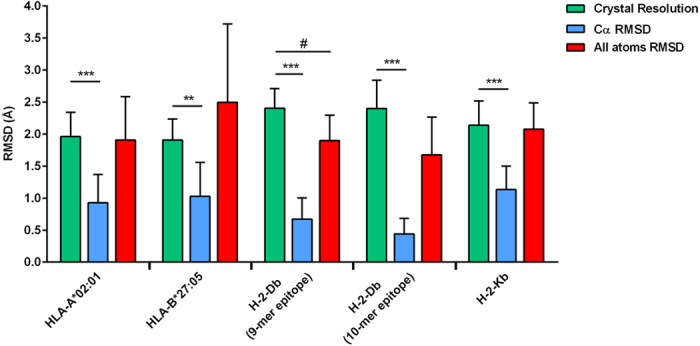
Graph with interleaved bars showing the mean Cα (blue) and all atoms (red) RMSD values in comparison to the resolution values extracted from the PDB (green), for each MHC-I allotype. The Cα RMSD mean value of all MHC-I allotypes was significantly below the crystal resolution mean values (***p < 0.0001, **p = 0.049). For H-2-Db (9-mer epitope), the all atoms RMSD mean value was also significantly below (#p = 0.0037). On y-axis, RMSD stands for Root Mean Square Deviation; on the x-axis, the MHC types are represented.

We retrieved from the PDB the crystal resolution value (in angstrom) of each structure analysed here and calculated the average. Using a Kolmogorov-Smirnov Test, we applied tests of normality to the crystal resolution, Cα RMSD and all atoms RMSD values for all allotypes. The HLA-A*02:01, HLA-B*27:05, H-2-Db (10-mer epitope) and H-2-Kb values were considered normally distributed (p = 0.200). Nevertheless, H-2-Db (9-mer epitope) values significantly deviated from normal distribution (p = 0.006). In this particular case, Kruskal-Wallis Test was performed. Each allotype was analysed individually (Fig. 3). It was observed that mean Cα RMSD values of DockTope validation were significantly below crystal resolution values for HLA-A*02:01 (p < 0.0001), HLA-B*27:05 (p = 0.049), H-2-Db (p < 0.0001) and H-2-Kb (p < 0.0001). Also, in the case of H-2-Db (9-mer epitope), the all atoms RMSD mean value was also significantly below the crystal resolution value (p = 0.0037).

### Web server

**Figure 4:**
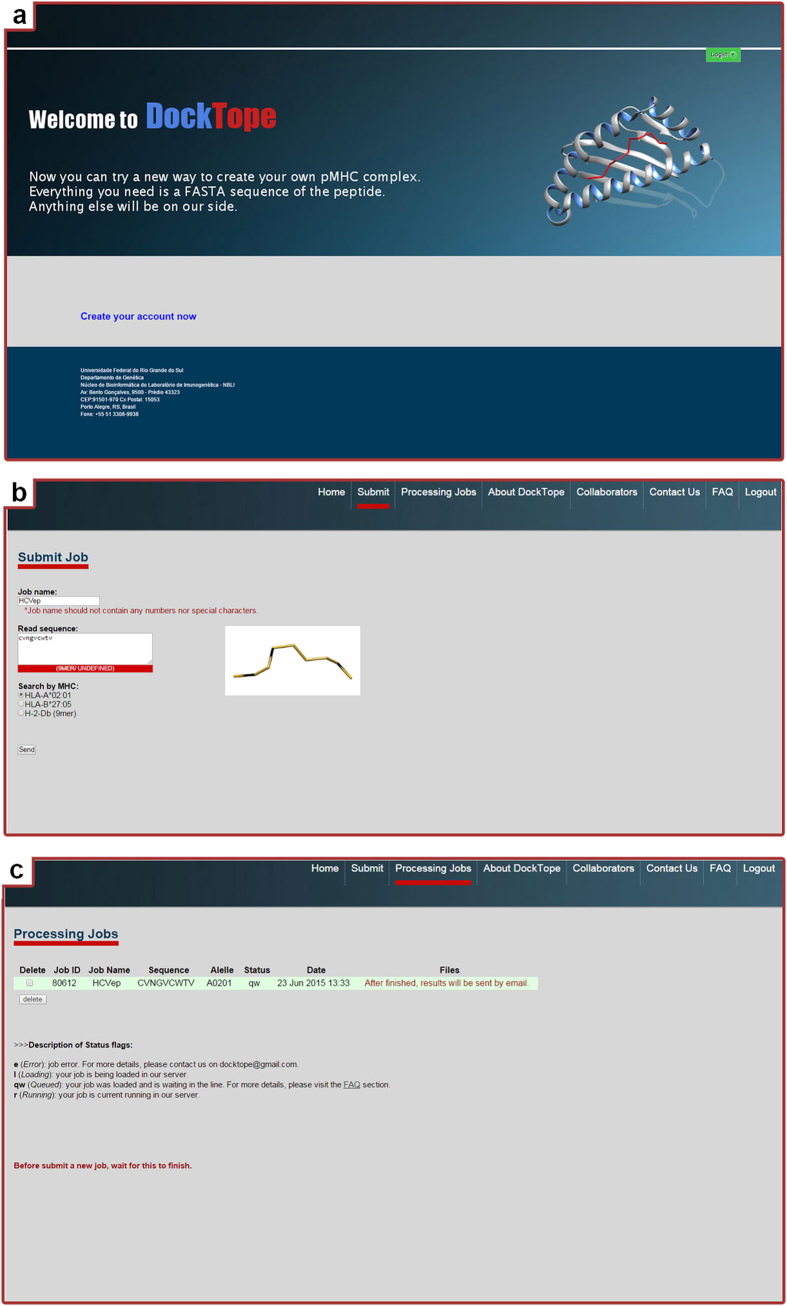
DockTope web interface. The first page the user will see is represented in (**a**), where it is possible to create a new account or directly access the tool with login and password. After the login, the user can submit a new sequence to be modeled, as represented in (**b**). Subsequently, the submitted job can be monitored through the “Processing jobs” tab (**c**).

DockTope is a freely accessible tool available through the website dirac.cesup.ufrgs.br/bio/home.php, or from the CrossTope platform (http://www.crosstope.com.br) under the ‘Tools’ tab^23^. First, the user should register an account. After that, the user receives an email with access data (login and password) to the site (Fig. 4a). To submit a new job, the user should provide a valid linear epitope sequence. The web server automatically recognizes the epitope sequence and provides a list with the possible MHC-I allotypes that can be used in the modelling (Fig. 4b). After submitting the job, the user can follow the process steps by clicking on the ‘Processing Jobs’ tab. A table is provided containing information about all jobs, such as Job ID, Job Name, Epitope sequence, MHC-I allele, Status, and Submission Date (Fig. 4c). After the job submission, a “Queued (qw)” flag is assigned. The time that the job stays with this flag will mainly depend on the demand. After that, the job proceeds to the “Running” state where the files are individually stored on our server. In case of error, the server stops the job. At the end of the process, a “Finalized” flag is assigned to the particular modelled epitope, and the pMHC-I structure file in the PDB format is sent to the registered email account provided by the user. The time spent on each job, after it enters the running state, will depend on the epitope sequence and allotype, though it should not exceed 6 hours. A 10-mer epitope of H-2-Db, for example, is expected to take longer, since the addition of one residue (in comparison with 9-mer epitopes) will increase dimensionality and require more computational time. It is also expected that epitopes with a large content of arginines, for example, take longer because of the increase in the number of side chain torsions.

The performance of the DockTope web server was assayed through the modelling of 238 immunogenic epitopes obtained from Immune Epitope Database and Analysis Resource^24^,^25^. In the end, 226 epitopes were modelled by DockTope without any error and 12 were aborted along the process-six of MHC-I allotype H-2-Db (9-mer epitope) and six of H-2-Db (10-mer epitope). These results represent an accuracy of approximately 95%.

## Discussion

In this work we described a fully automated tool for the structural prediction of peptide:MHC-I (pMHC-I) complexes, DockTope, which was developed and validated for the MHC-I allotypes HLA-A*02:01, HLA-B*27:05, H-2-Kb and H-2-Db. DockTope was able to reproduce 135 crystal structures from the PDB with a RMSD mean value of 0.882 Å and 1.964 Å, for epitope Cα and all atoms, respectively. The final accomplishment of this tool is (i) the complete automation, first presented here, of the established approach D1-EM-D2^18^, (ii) the modelling validation of all non-redundant pMHC-I structures available in the PDB at this time, and (iii) the tool availability as a web server for any researcher or user interested in pMHC-I modelling.

To automate and validate DockTope, a specific bash script was developed and executed in each step of the pMHC-I complex construction. The structure validation was performed using each pMHC-I structure in the PDB as a target to calculate the RMSD value with its respective model. Target and model were always fitted by MHC-I residues, which ensures that not only the difference between each epitope residue pair is considered, but also its displacement inside the MHC-I cleft after the molecular docking/energy minimization process. It should be noted that molecular docking programs can find unusual conformations after the searching process. To avoid this, DockTope performs a total of 20 rounds of molecular docking, generating up to 1000 conformations, which increases the probability of finding a proper epitope conformation. Still, unusual conformations can be generated (such as an inverted epitope inside the cleft or a protuberant C-terminal/N-terminal extremity pointing outside the MHC-I cleft). This phenomenon can be biologically explained, since some MHC-I allotypes do not have the capability to interact with determined epitope residues, but it can also be simply due to the fact that the docking algorithm was unable to find the correct solution. From the biological point of view, a work developed by Sidney *et al.* encompassing 945 HLA-A and HLA-B molecules reveals that some physicochemical specificities are not found in the evaluated MHC-I allotypes (considering B and F pocket residues), which in turn prevents the binding and presentation of peptides with such features^26^. In order to avoid a misleading result, DockTope also automatically checks the epitope position and orientation (but not the binding affinity) after molecular docking, confirming its position inside the MHC-I cleft before proceeding to the search for the best pMHC-I structure. Of note, this is one of the most common sources of error reported by DockTope.

Before the implementation and automation of DockTope, the best pMHC-I structure was chosen through visual inspection only, where the most frequent conformation among the twenty generated was selected. This way, a user intervention was required, which could bias the result. Here, a new and improved algorithm is used to choose the best structure, based on the mean RMSD value between each epitope pair and on the binding energy value generated by AutoDock Vina.

Since there is a lack of pMHC-I crystal structures available in the PDB, our analysis was restricted to MHC-I allotype H-2-Kb (8-mer epitope), HLA-A*02:01 (9-mer epitope), HLA-B*27:05 (9-mer epitope), and H-2-Db (9-mer epitope and 10-mer epitope). This ensures that only experimentally-resolved protein structures are used to identify the MHC-I allotype-specific epitope pattern, which reinforces the technique specificity. Also, the low number of MHC-I allotypes available for modelling by DockTope should not be seen as a weakness, since it opens the theoretical possibility to model roughly 1.2 × 1013 pMHC-I structures, which would be unfeasible through X‐ray crystallography or any other method currently available. Moreover, the importance of each one of these allotypes should be highlighted. The HLA-A*02 molecule is expressed by approximately half of the human population, and the HLA-A*02:01 allele is found in a relatively high frequency all over the world^27^. For this reason, it is one of the most studied alleles. The HLA-B*27:05 has been associated with spondyloarthropathies disorders, such as ankylosing spondylitis^28^,^29^,^30^, vaccine response^31^,^32^, and HIV in elite controllers^33^,^34^. The H-2-Db and H-2-Kb are widely-studied murine alleles, and recent studies have demonstrated its importance in synapse pruning of developing brain in murines^35^,^36^.

Regarding the RMSD values for the DockTope validation (see Table 2 and Fig. 2), the overall RMSD average for all modelled epitopes, considering Cα and all atoms, remained below 2 Å; this is considered a reference cut-off value indicating a valid crystal reproduction obtained through a cross-docking approach^19^,^37^,^38^,^39^. In fact, the validation values are reinforced after the comparison of the Cα and all atoms RMSD values by the average resolution value extracted from the PDB for all 135 structures analysed in this work (Fig. 3). The crystal resolution average value of the reproduced dataset, considering all allotypes, was 2.163 Å, which is higher than the RMSD value obtained for Cα and all atoms (0.839 Å and 2.012 Å, respectively). Analysing each pMHC-I individually, we observed that all Cα RMSD mean values were significantly below the crystal resolution mean. This indicates that subtle deviations between target and model are expected, especially because the epitope is not a rigid body inside the MHC-I cleft, and normal amino acid fluctuations can occur^22^. It came to our attention that only HLA-B*27:05-restricted complexes presented all atoms RMSD values greater than the respective crystal resolution average. This is attributed to the fact that HLA-B*27:05-restricted epitopes present a high proportion of arginine residues^40^, containing long side chains, which in turn accounts for most of the RMSD deviation observed.

**Figure 5:**
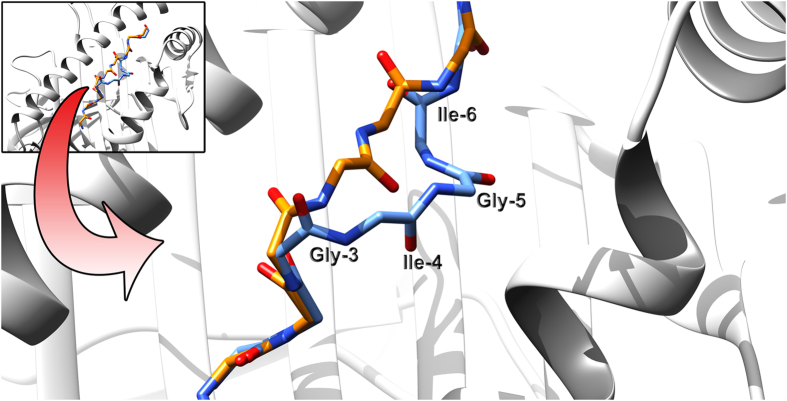
Epitope backbone comparison between the target (PDB ID: 2GTW), in blue, and the reproduced model, in orange, in the context of HLA-A*02:01. A top view of the pMHC-I is shown in the upper left. The arrow indicates the region (expanded at the centre) where most of the Cα RMSD is observed, which accounts for a high value of 3.129 Å.

Most of the Cα RMSD data was distributed below 1.5 Å, indicating a high precision of our technique (Fig. 2a). However, we observed an incoherent value of 3.129 Å for one of the epitopes bound to HLA-A*02:01. This value corresponds to the epitope LAGIGILTV derived from the MART-1/Melan-A protein (PDB ID: 2GTW). This epitope represents a variant of the 10-mer epitope **E**LAGIGILTV, which is recognized by MART-1-reactive T cells^41^. The interesting fact is that this 10-mer epitope presents a bulged conformation comprising the residues Gly-Ile-Gly-Ile, which is replicated by the 9-mer epitope LAGIGILTV, comprising the same residues. To adopt this conformation, the P1 leucine residue of the 9-mer epitope is inserted into the P2 pocket, exactly as it occurs with the 10-mer epitope^42^. This bulged conformation accounts for the major deviation values observed between the model and the crystal structure (Fig. 5). As discussed by Borbulevych *et al.*, this bulged conformation differs from other HLA-A*02:01 bound 9-mer epitopes from MART-1/Melan-A protein, such as ALGIGILTV (PDB ID: 2GTZ) and AAGIGILTV (PDB ID: 3QFD), which present a common extended conformation and incidentally produced better Cα RMSD values here (1.435 Å and 1.377 Å, respectively). It is important to note that DockTope is based on a technique that uses epitope backbone patterns inside the MHC-I cleft; thus it is possible that unusual or aberrant epitope conformations will not be properly assessed.

**Figure 6:**
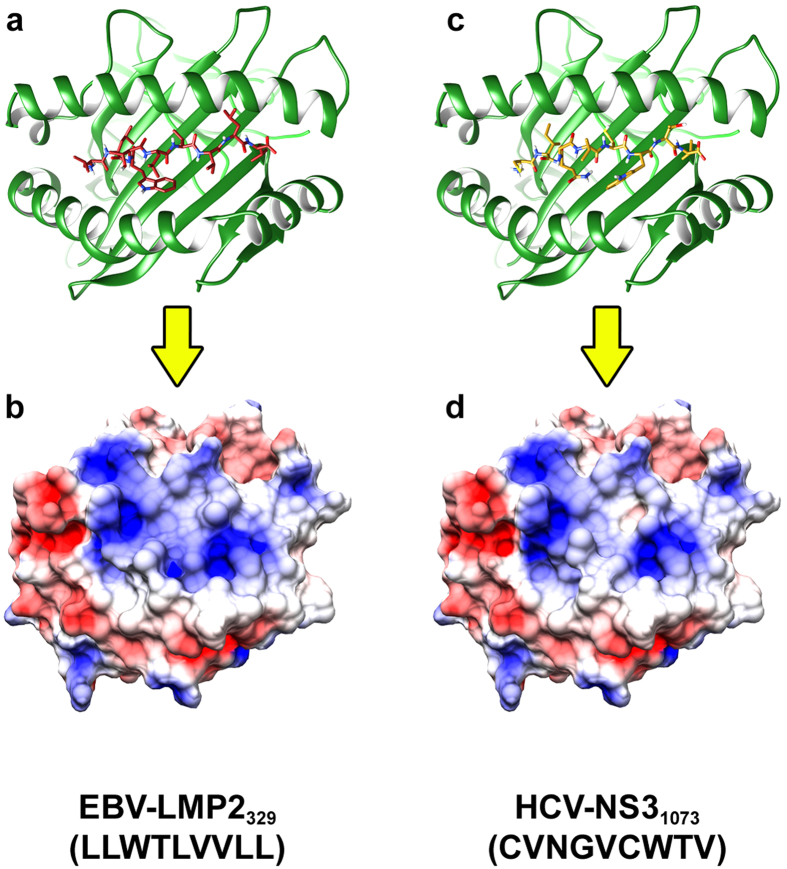
Two pMHC-I structures modelled using DockTope. In (**a**) and (**c**), the MHC-I (ribbon representation) and the epitope (stick representation) are depicted. In (**b**) and (**d**), the molecular surface of the TCR-interacting area was computed using UCSF Chimera package from the Computer Graphics Laboratory^50^,^51^ and the electrostatic potential was calculated using DelPhi^52^. The colour range (−3 kT to +3 kT, where *k* represents the Boltzmann constant and *T* represents the temperature) indicates the positive (blue), neutral (white) and negative (red) charges distributed on the pMHC-I surface. In (a) and (b), the epitope EBV-LMP2_329_ (LLWTLVVLL) is represented and in (c) and (d) the epitope HCV-NS3_1073_ (CVNGVCWTV) is represented.

Structures generated using DockTope can be used in several immunology fields, such as cancer research, transplantation, *in silico* stabilization assays and cross-reactivity assessment, expanding the range of possibilities to study these topics. In fact, our tool have already proven to be useful when studying cross-reactivity among different pMHC-I complexes. In a previous work, Principal Component Analysis (PCA) and Hierarchical Clustering Analysis (HCA) were employed to compare electrostatic potential data of TCR-interacting residues presented on the pMHC-I surface^43^. A total of 28 known HCV targets (epitopes from NS3 protein) were modelled and analysed. The differences observed in PCA and HCA were evidences for structure-dependent immunogenic patterns and were in accordance with *in vitro* data of IFN-γ releasing assays^44^. After that, 55 pMHC-I complexes including epitopes from different viral proteins were also modelled; this allowed us to infer other potentially cross-reactive targets with HCV-NS3_1073_, such as LMP2_329_ from Epstein-Barr virus (EBV), Gag_77_ from Human Immunodeficiency virus (HIV), and NA_231_ from Influenza virus (IV). Of note, cross-reactive responses of NS3_1073_-specific CD8^ + ^T cells against all of these targets were later confirmed through *in vitro* assays^45^. Intriguingly, the linear sequence of the confirmed cross-reactive epitope EBV-LMP2_329_ presents no similarities in amino acid sequence with the reference HCV-NS3_1073_ epitope, and shares only 33% of biochemical properties. Sequence-based analysis would most likely be unable to predict such cross-reactivity. However, an incredible resemblance is observed in a higher level of complexity, through the analysis of the TCR-interacting surface of the pMHC-I. Such analysis was made possible by modelling these pMHC-I complexes through D1-EM-D2, the approach behind DockTope (Fig. 6).

Three approaches stand out among previously published methodologies aiming at the pMHC structural prediction: (i) MHCsim^10^, (ii) pDOCK^8^, and (iii) a Biased-Probability Monte Carlo docking protocol published by Bordner and Abagyan^9^. MHCsim was the first automated server designed to model pMHC complexes. The server uses the input sequences (MHC and epitope) to perform a search in an internal database for the pMHC structure that is the most similar to the input sequences. Then, the template is modified at the positions where the residues differ to generate a new 3D structure. Some aspects not included in MHCsim are addressed by DockTope. The MHCsim methodology is based only on sequence similarity, which might not be sufficiently accurate to predict the 3D structure of a pMHC complex, especially when it comes to epitope conformation. This way, the provided pMHC structure is not final, but can be used for posterior refinement^46^. Second, the MHCsim server allows the pMHC construction for human allotypes only, and is restricted to 9-mer epitopes. The pDOCK methodology is based mainly on ICM docking, Monte Carlo sampling and local minimization. In its validation, the authors presented Cα RMSD values below 1 Å in a set of 186 pMHC-I and pMHC-II structures. However, contrary to DockTope, which used cross-docking to reproduce crystal structures, the validation process of pDOCK was performed through a re-docking approach and all atom RMSD values were not provided in the text. Also, pDOCK is currently not available as a web server, but only as an in-house protocol. The method published by Bordner and Abagyan is based on ICM docking, homology modelling and Support Vector Machine (SVM). They were able to reproduce through cross-docking a set of 14 HLA-A*02:01 epitopes and 9 H-2-Kb epitopes with epitope backbone RMSD values inferior to 1 Å. Like pDOCK, their method is not available as a web server.

DockTope emerges as a free, automated, well-validated (Cα RMSD mean values below 1 Å), and user-friendly web-server tool for modelling pMHC-I complexes in a reliable way. Its usefulness was already demonstrated by previously published work. The possibility to construct pMHC-I complexes will open new avenues for structural immunoinformatics, hopefully triggering new discoveries in basic immunology and health applied sciences.

## Methods

### DockTope Automation

DockTope was developed as an optimized tool based on the D1-EM-D2 pMHC-I modelling approach. In order to automate the process, we employed a series of 9 shell scripts, 13 python scripts, 7 C++ executables and 2 python executables to perform the following steps: (i) Epitope structure modelling, (ii) first molecular docking (D1), (iii) choosing the best structure from D1, (iv) second molecular docking (D2), (v) choosing the best structure from D2 and (vi) writing the output. These steps are represented in the flowchart of Fig. 1 and in Supplementary Fig. S1.

### Epitope structure modelling

The epitope to be modelled is provided as a linear amino acid sequence (without three-dimensional coordinates). A python script, which launches a built-in PyMOL^47^ plug-in, uses the backbone of the epitope pattern to give shape to the modelled epitope. This epitope undergoes energy minimization, allowing for a mild global relaxation of the peptide.

### First (D1) and Second (D2) Molecular Docking

Molecular docking is performed using the programs AutoDock Tools^48^ and AutoDock Vina^19^; it involves three main steps. In the first step, the MHC-I molecule is prepared according to the following protocol: (i) adding all hydrogens, (ii) adding Gasteiger charges and (iii) removing non-polar hydrogens. In the second step, the modelled epitope is prepared by repeating the same protocol used for the MHC-I, but including an additional level: the torsion tree is set in a manner to maintain the epitope backbone rigid during the molecular docking process, thus only allowing the movement of side chains. In the final step, a box grid is configured to allow the search for the best epitope conformations inside the MHC-I cleft (formed by the α1 and α2 domains). The search for the best conformation is performed according to specific algorithms^19^ along twenty rounds (arbitrary value). In the end, the best structure is chosen according to an algorithm developed by our group.

### Energy Minimization (EM)

The energy minimization process is performed using the GROMACS package^21^. This process is used twice: on the modelled epitope and on the best pMHC-I complex produced by the first molecular docking, with the final goal of removing possible steric clashes and correcting distances between atoms in the system. The EM protocol is performed using a virtual cubic box filled with the protein and water (Simple Point Charge water model). Ions Na^+^ and Cl^−^ are included to neutralize the system, maintaining a final concentration of 0.15 M/L. The GROMOS53a5 force field^49^ is used to compute inter- and intramolecular interactions. The cut-off distances for the Coulomb (electrostatic and long-range attraction) and Lennard-Jones (repulsion and short-range attraction) forces are set to 1 nm. Molecular dynamics parameters include the steepest descent method of integration, with no constraints and a total of 10,000 steps, with an initial time step of 0.001 nm. The minimization converges after the maximum force is smaller than 2,000 kJ mol^−1^ nm^−1^; the lowest energy coordinates are then written to a file.

### Choosing the best structure

The output of the first and second molecular docking process is composed of the best 20 epitope conformations in a set that could contain up to 1000 conformations. The best conformation, among these twenty, is chosen according to the following equations:


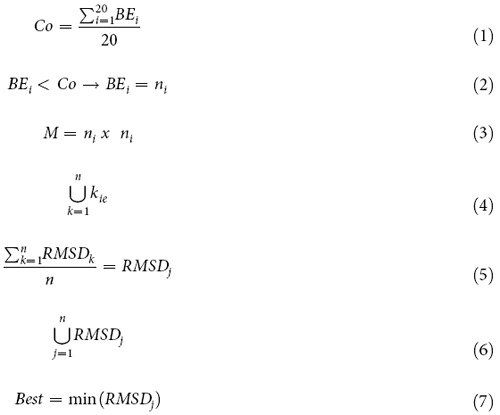


Where, *BE* represents the binding energy value provided by AutoDock Vina for each epitope conformation (i = 1, i = 2,…, i = 20); *Co* represents a cut-off value based on the average of the twenty BE; “*n*” represents the structure conformation chosen based on equation (2) and that will be used in the next steps of the calculation; “*M”* represents a matrix used to combine the selected data from equation (2) “*k*_*ie*_” is the resultant file from the union (“*U*”) of all data from equation (3); “*RMSD*_*j*_” represents the file containing the RMSD average from the data contained in the *k*_*ie*_ files; and “*Best*” is the final structure containing the three-dimensional coordinates of the chosen epitope.

### DockTope validation

For each pMHC-I structure downloaded from the PDB, the epitope and MHC-I parts were separated. Next, the epitope linear sequence and the MHC-I allele name (according to Table 1) were used as input for a cross-docking process, using DockTope. At the end of the process, the modelled pMHC-I structure was compared to its respective structure available in the PDB; quantitative data was obtained through the RMSD analysis of the two structures, considering the Cα and the all atoms RMSD displacement of the epitope. For the analysis, the structures available in the PDB were refined to contain only the pMHC-I structure, without TCR and possible ligands interfering with the peptide:MHC-I interaction. In the end, a total of 135 pMHC-I structures encompassing the MHC-I allotypes HLA-A*02:01, HLA-B*27:05, H-2-Db and H-2-Kb were evaluated (Table 2). The performance of DockTope was also evaluated through the modelling of 238 epitopes downloaded from the Immune Epitope Database and Analysis Resource (IEDB). The IEDB parameters for epitope search were set to contain only linear epitopes, from any disease, and confirmed by T cell assays (positive).

### Web server

DockTope can be accessed through the CrossTope website (http://www.crosstope.com.br), in the “Tools” tab, or directly from dirac.cesup.ufrgs.br/bio/home.php. To use this tool, the user should sign in providing basic information such as name, email address, institution and academic degree. After logging in, the user will find the following tabs: Home, Submit, Processing Jobs, About DockTope, Collaborators, Contact Us and Frequent Asked Questions (FAQ). The web server includes two interfaces: user-tool and tool-server. The user-tool interface uses more than one programming language to better integrate all the modules. The visual module (web interface) was developed in PHP and jQuery Ajax, which are based on an HTML structure. All internal actions of the web interface are controlled and executed through JavaScript, especially processes validation, such as login validation, for example. The interface management and integration service available to the user, as well as the non-visible part (such as the execution of .js files) were obtained through the XAMPP server, which includes the APACHE, MySQL and PHP packages. The tool-server interface works exclusively through JavaScript (connection, submission and receipt of submitted jobs). A verification module works constantly over each created page to ensure the database connection, allowing access to the user. All the jobs, after being submitted, enter in a queue until the server checks and allows them to run.

### Statistical analysis

The statistical analyses were performed using SPSS Software (IBM SPSS Statistics for Windows, Version 16.0. Armonk, NY: IBM Corp) and GraphPad Prism version 6.05 for Windows (GraphPad Software, La Jolla California USA, www.graphpad.com). We checked the normality of the data with the Kolmogorov-Smirnov Test, considering a level of significance of 0.05 (p < 0.05). We used one-way analysis of variance (one-way ANOVA) to perform multiple comparisons of the averages. The statistic of normal distribution data was analysed with Tukey’s post-hoc test. Data without normal distribution was analysed with the Kruskal-Wallis test.

## Additional Information

**How to cite this article**: Menegatti Rigo, M. *et al.* DockTope: a Web-based tool for automated pMHC-I modelling. *Sci. Rep.* **5**, 18413; doi: 10.1038/srep18413 (2015).

## Supplementary Material

Supplementary Information

## Figures and Tables

**Table 1:** PDB structures (MHC-I and epitope) used by DockTope.

	PDB ID (Resolution)		
Allotype	MHC-I	Epitope	Reference
HLA-A*02:01	2V2W (1.6 Å)	1T1Z (1.9 Å) (ALYNTAAAL)	53,54
HLA-B*27:05	2A83 (1.4 Å)	1JGE (2.1 Å) (GRFAAAIAK)	55,56
H-2-Db	1WBX (1.9 Å)	1JPG (2.2 Å, 9-mer epitope) (FQPQNGQFI)	57,58
H-2-Db	1WBY (2.3 Å)	1WBY (2.3 Å, 10-mer epitope) (SSLENFRAYV)	57
H-2-Kb	1LK2 (1.35 Å)	1RJY (1.9 Å) (SSIEFARL)	59,60

**Table 2:** DockTope validation based on Cα and all atoms RMSD average values, in angstroms.

			RMSD (Cα)		RMSD (all-atom)	
MHC-I allotype	Epitope Length	Number of pMHC-I structures	Mean	s.d. (s.e.m)	Mean	s.d. (s.e.m)
HLA-A*02:01	9	68	0.926 Å	± 0.440 (0.053)	1.908 Å	± 0.678 (0.082)
HLA-B*27:05	9	10	1.027 Å	± 0.530 (0.167)	2.498 Å	± 1.224 (0.387)
H-2-Db	9	33	0.671 Å	± 0.331 (0.057)	1.899 Å	± 0.396 (0.069)
H-2-Db	10	5	0.439 Å	± 0.244 (0.109)	1.676 Å	± 0.590 (0.264)
H-2-Kb	8	19	1.132 Å	± 0.365 (0.083)	2.077 Å	± 0.412 (0.094)
**TOTAL**		**135**	**0.882 Å**	** ± 0.437 (0.037)**	**1.964 Å**	** ± 0.655 (0.056)**

The standard deviation (s.d.) and the standard error of the mean (s.e.m.) are also provided.

## References

[b1] Yewdell J. W. & Bennink J. R. Immunodominance in major histocompatibility complex class I-restricted T lymphocyte responses. Annu Rev Immunol 17, 51–88 (1999). 10358753 10.1146/annurev.immunol.17.1.51

[b2] Jorgensen J. L., Esser U., Fazekas de St Groth B., Reay P. A. & Davis M. M. Mapping T-cell receptor-peptide contacts by variant peptide immunization of single-chain transgenics. Nature 355, 224–230 (1992). 1309938 10.1038/355224a0

[b3] Wooldridge L. *et al.* A single autoimmune T cell receptor recognizes more than a million different peptides. J Biol Chem 287, 1168–1177 (2012). 22102287 10.1074/jbc.M111.289488 PMC3256900

[b4] He L. *et al.* Integrated assessment of predicted MHC binding and cross-conservation with self reveals patterns of viral camouflage. BMC Bioinformatics 15, (Suppl 4):S1 (2014). 10.1186/1471-2105-15-S4-S1 PMC4094998 25104221

[b5] Mungall A. J. *et al.* The DNA sequence and analysis of human chromosome 6. Nature 425, 805–811 (2003). 14574404 10.1038/nature02055

[b6] Park H. J., Kim J. Y., Jung K. I. & Kim T. J. Characterization of a Novel Gene in the Extended MHC Region of Mouse, NG29/Cd320, a Homolog of the Human CD320. Immune Netw 9, 138–146 (2009). 20157601 10.4110/in.2009.9.4.138 PMC2816947

[b7] Berko D. *et al.* Membrane-anchored beta 2-microglobulin stabilizes a highly receptive state of MHC class I molecules. J Immunol 174, 2116–2123 (2005). 15699142 10.4049/jimmunol.174.4.2116

[b8] Khan J. M. & Ranganathan S. pDOCK: a new technique for rapid and accurate docking of peptide ligands to Major Histocompatibility Complexes. Immunome Research 6, (Suppl 1):S2 (2010). 10.1186/1745-7580-6-S1-S2 PMC2946780 20875153

[b9] Bordner A. J. & Abagyan R. Ab initio prediction of peptide-MHC binding geometry for diverse class I MHC allotypes Proteins 63, 512–526 (2006). 16470819 10.1002/prot.20831

[b10] Todman S. J. *et al.* Toward the atomistic simulation of T cell epitopes automated construction of MHC: peptide structures for free energy calculations. J Mol Graph Model 26, 957–961 (2008). 17766153 10.1016/j.jmgm.2007.07.005

[b11] Schafroth H. D. & Floudas C. A. Predicting peptide binding to MHC pockets via molecular modeling, implicit solvation, and global optimization. Proteins 54, 534–556 (2004). 14748001 10.1002/prot.10608

[b12] Tong J. C., Tan T. W. & Ranganathan S. Modeling the structure of bound peptide ligands to major histocompatibility complex. Protein Sci 13, 2523–2532 (2004). 15322290 10.1110/ps.04631204 PMC2279999

[b13] Rognan D., Lauemoller S. L., Holm A., Buus S. & Tschinke V. Predicting binding affinities of protein ligands from three-dimensional models: application to peptide binding to class I major histocompatibility proteins. J Med Chem 42, 4650–4658 (1999). 10579827 10.1021/jm9910775

[b14] Sezerman U., Vajda S. & DeLisi C. Free energy mapping of class I MHC molecules and structural determination of bound peptides. Protein Sci 5, 1272–1281 (1996). 8819160 10.1002/pro.5560050706 PMC2143467

[b15] Rosenfeld R., Zheng Q., Vajda S. & DeLisi C. Computing the structure of bound peptides. Application to antigen recognition by class I major histocompatibility complex receptors. J Mol Biol 234, 515–521 (1993). 8254656 10.1006/jmbi.1993.1607

[b16] Antes I. DynaDock: A new molecular dynamics-based algorithm for protein-peptide docking including receptor flexibility. Proteins 78, 1084–1104 (2009). 20017216 10.1002/prot.22629

[b17] Antes I., Siu S. W. & Lengauer T. DynaPred: a structure and sequence based method for the prediction of MHC class I binding peptide sequences and conformations. Bioinformatics 22, e16–24 (2006). 16873467 10.1093/bioinformatics/btl216

[b18] Antunes D. A. *et al.* Structural allele-specific patterns adopted by epitopes in the MHC-I cleft and reconstruction of MHC:peptide complexes to cross-reactivity assessment. PLoS One 5, e10353 (2010). 20442757 10.1371/journal.pone.0010353 PMC2860844

[b19] Trott O. & Olson A. J. AutoDock Vina: improving the speed and accuracy of docking with a new scoring function, efficient optimization, and multithreading. Journal of Computational Chemistry 31, 455–461 (2009). 19499576 10.1002/jcc.21334 PMC3041641

[b20] Zhou H. X. & Gilson M. K. Theory of free energy and entropy in noncovalent binding. Chem Rev 109, 4092–4107 (2009). 19588959 10.1021/cr800551w PMC3329805

[b21] Van Der Spoel D. *et al.* GROMACS: fast, flexible, and free. Journal of Computational Chemistry 26, 1701–1718 (2005). 16211538 10.1002/jcc.20291

[b22] Reboul C. F., Meyer G. R., Porebski B. T., Borg N. A. & Buckle A. M. Epitope flexibility and dynamic footprint revealed by molecular dynamics of a pMHC-TCR complex. PLoS Comput Biol 8, e1002404 (2012). 22412359 10.1371/journal.pcbi.1002404 PMC3297556

[b23] Sinigaglia M., Antunes D. A., Rigo M. M., Chies J. A. & Vieira G. F. CrossTope: a curate repository of 3D structures of immunogenic peptide: MHC complexes. Database (Oxford) 2013, bat002 (2013). 23396301 10.1093/database/bat002 PMC3567486

[b24] Vita R. *et al.* *Immune Epitope Database and Analysis ResourceI.* (2015) Available at: www.iedb.org. (Accessed: 6th October 2015)

[b25] Vita R. *et al.* The immune epitope database (IEDB) 3.0. Nucleic Acids Res 43, D405–412 (2014). 25300482 10.1093/nar/gku938 PMC4384014

[b26] Sidney J., Peters B., Frahm N., Brander C. & Sette A. HLA class I supertypes: a revised and updated classification. BMC Immunology 9, 1 (2008). 18211710 10.1186/1471-2172-9-1 PMC2245908

[b27] Choo J. A., Liu J., Toh X., Grotenbreg G. M. & Ren E. C. The immunodominant influenza A virus M158-66 cytotoxic T lymphocyte epitope exhibits degenerate class I major histocompatibility complex restriction in humans. J Virol 88, 10613–10623 (2014). 24990997 10.1128/JVI.00855-14 PMC4178881

[b28] Nasution A. R. *et al.* HLA-B27 subtypes positively and negatively associated with spondyloarthropathy. J Rheumatol 24, 1111–1114 (1997). 9195518

[b29] Abualrous E. T. *et al.* F pocket flexibility influences the tapasin dependence of two differentially disease-associated MHC Class I proteins. Eur J Immunol 45, 1248–1257 (2015). 25615938 10.1002/eji.201445307

[b30] Powis S. J., Santos S. G. & Antoniou A. N. Biochemical features of HLA-B27 and antigen processing. Adv Exp Med Biol 649, 210–216 (2009). 19731631 10.1007/978-1-4419-0298-6_15

[b31] Posteraro B. *et al.* The link between genetic variation and variability in vaccine responses: systematic review and meta-analyses. Vaccine 32, 1661–1669 (2014). 24513009 10.1016/j.vaccine.2014.01.057

[b32] Ovsyannikova I. G., Pankratz V. S., Larrabee B. R., Jacobson R. M. & Poland G. A. HLA genotypes and rubella vaccine immune response: additional evidence. Vaccine 32, 4206–4213 (2014). 24837503 10.1016/j.vaccine.2014.04.091 PMC4124933

[b33] Loffredo J. T. *et al.* Two MHC class I molecules associated with elite control of immunodeficiency virus replication, Mamu-B*08 and HLA-B*2705, bind peptides with sequence similarity. J Immunol 182, 7763–7775 (2009). 19494300 10.4049/jimmunol.0900111 PMC2701622

[b34] Kaslow R. A. *et al.* Influence of combinations of human major histocompatibility complex genes on the course of HIV-1 infection. Nat Med 2, 405–411 (1996). 8597949 10.1038/nm0496-405

[b35] Adelson J. D. *et al.* Developmental Sculpting of Intracortical Circuits by MHC Class I H2-Db and H2-Kb. Cereb Cortex, 1–11 (2014). 10.1093/cercor/bhu243 PMC4785944 25316337

[b36] Lee H. *et al.* Synapse elimination and learning rules co-regulated by MHC class I H2-Db. Nature 509, 195–200 (2014). 24695230 10.1038/nature13154 PMC4016165

[b37] Bergeron B. Bioinformatics Computing 1st edn (Prentice Hall, 2002).

[b38] Bagaria A., Jaravine V., Huang Y. J., Montelione G. T. & Guntert P. Protein structure validation by generalized linear model root-mean-square deviation prediction. Protein Sci 21, 229–238 (2011). 22113924 10.1002/pro.2007 PMC3324767

[b39] Madurga S., Belda I., Llora X. & Giralt E. Design of enhanced agonists through the use of a new virtual screening method: application to peptides that bind class I major histocompatibility complex (MHC) molecules. Protein Sci 14, 2069–2079 (2005). 16046628 10.1110/ps.051351605 PMC2279318

[b40] Madden D. R., Gorga J. C., Strominger J. L. & Wiley D. C. The structure of HLA-B27 reveals nonamer self-peptides bound in an extended conformation. Nature 353, 321–325 (1991). 1922337 10.1038/353321a0

[b41] Kawakami Y. *et al.* Identification of the immunodominant peptides of the MART-1 human melanoma antigen recognized by the majority of HLA-A2-restricted tumor infiltrating lymphocytes. J Exp Med 180, 347–352 (1994). 7516411 10.1084/jem.180.1.347 PMC2191573

[b42] Borbulevych O. Y. *et al.* Structures of MART-126/27-35 Peptide/HLA-A2 complexes reveal a remarkable disconnect between antigen structural homology and T cell recognition. J Mol Biol 372, 1123–1136 (2007). 17719062 10.1016/j.jmb.2007.07.025 PMC2134917

[b43] Antunes D. A. *et al.* Structural in silico analysis of cross-genotype-reactivity among naturally occurring HCV NS3-1073-variants in the context of HLA-A*02:01 allele. Molecular Immunology 48, 1461–1467 (2011). 21513985 10.1016/j.molimm.2011.03.019

[b44] Fytili P. *et al.* Cross-genotype-reactivity of the immunodominant HCV CD8 T-cell epitope NS3-1073. Vaccine 26, 3818–3826 (2008). 18582999 10.1016/j.vaccine.2008.05.045

[b45] Zhang S. *et al.* Frequency, private specificity and cross-reactivity of pre-existing HCV-specific CD8 + T cells in HCV seronegative individuals: implication for vaccine responses. J Virol 89, 8304–8317 (2015). 26041301 10.1128/JVI.00539-15 PMC4524240

[b46] Vivona S. *et al.* Computer-aided biotechnology: from immuno-informatics to reverse vaccinology. Trends Biotechnol 26, 190–200 (2008). 18291542 10.1016/j.tibtech.2007.12.006

[b47] Schrodinger L. L. C. The PyMOL Molecular Graphics System, Version 1.3r1 (2010).

[b48] Morris G. M. *et al.* AutoDock4 and AutoDockTools4: Automated docking with selective receptor flexibility. Journal of Computational Chemistry 30, 2785–2791 (2009). 19399780 10.1002/jcc.21256 PMC2760638

[b49] Oostenbrink C., Villa A., Mark A. E. & van Gunsteren W. F. A biomolecular force field based on the free enthalpy of hydration and solvation: the GROMOS force-field parameter sets 53A5 and 53A6. Journal of Computational Chemistry 25, 1656–1676 (2004). 15264259 10.1002/jcc.20090

[b50] Pettersen E. F. *et al.* UCSF Chimera—a visualization system for exploratory research and analysis. J Comput Chem 25, 1605–1612 (2004). 15264254 10.1002/jcc.20084

[b51] Sanner M. F., Olson A. J. & Spehner J. C. Reduced surface: an efficient way to compute molecular surfaces. Biopolymers 38, 305–320 (1996). 8906967 10.1002/(SICI)1097-0282(199603)38:3%3C305::AID-BIP4%3E3.0.CO;2-Y

[b52] Li L. *et al.* DelPhi: a comprehensive suite for DelPhi software and associated resources. BMC Biophys 5, 9 (2012). 22583952 10.1186/2046-1682-5-9 PMC3463482

[b53] Lee J. K. *et al.* T cell cross-reactivity and conformational changes during TCR engagement. J Exp Med 200, 1455–1466 (2004). 15583017 10.1084/jem.20041251 PMC2211951

[b54] Martinez-Hackert E. *et al.* Structural basis for degenerate recognition of natural HIV peptide variants by cytotoxic lymphocytes. J Biol Chem 281, 20205–20212 (2006). 16702212 10.1074/jbc.M601934200

[b55] Ruckert C. *et al.* Conformational dimorphism of self-peptides and molecular mimicry in a disease-associated HLA-B27 subtype. J Biol Chem 281, 2306–2316 (2006). 16221670 10.1074/jbc.M508528200

[b56] Hulsmeyer M. *et al.* HLA-B27 subtypes differentially associated with disease exhibit subtle structural alterations. J Biol Chem 277, 47844–47853 (2002). 12244049 10.1074/jbc.M206392200

[b57] Meijers R. *et al.* Crystal structures of murine MHC Class I H-2 D(b) and K(b) molecules in complex with CTL epitopes from influenza A virus: implications for TCR repertoire selection and immunodominance. J Mol Biol 345, 1099–1110 (2005). 15644207 10.1016/j.jmb.2004.11.023

[b58] Ciatto C. *et al.* Zooming in on the hydrophobic ridge of H-2D(b): implications for the conformational variability of bound peptides. J Mol Biol 312, 1059–1071 (2001). 11580250 10.1006/jmbi.2001.5016

[b59] Rudolph M. G. *et al.* A peptide that antagonizes TCR-mediated reactions with both syngeneic and allogeneic agonists: functional and structural aspects. J Immunol 172, 2994–3002 (2004). 14978103 10.4049/jimmunol.172.5.2994

[b60] Miley M. J. *et al.* Structural basis for the restoration of TCR recognition of an MHC allelic variant by peptide secondary anchor substitution. J Exp Med 200, 1445–1454 (2004). 15557346 10.1084/jem.20040217 PMC2211956

