# Supplementary material for: DockTope: a Web-based tool for automated pMHC-I modelling
Source: Sci Rep. 2015 Dec 17;5:18413. doi: 10.1038/srep18413 (PMC4682062; doi:10.1038/srep18413)
Supplement: Supplementary Information [file srep18413-s1.pdf]

DockTope: a Web-based tool for automated pMHC-I modelling.

Maurício Menegatti Rigo,

Dinler Amaral Antunes,

Martiela Vaz de Freitas,

Marcus Fabiano de Almeida Mendes,

Lindolfo Meira,

Marialva Sinigaglia,

Gustavo Fioravanti Vieira.

Supplementary Figure S1. pMHC-I complex construction process employed by DockTope. In (A), the MHC donor and the epitope pattern were obtained for each allotype (Table 1 of the manuscript for details). In (B), the full automated process. The automation follows a sequence of steps: (a) structural alignment between the target and the modeled epitope, (b) first molecular docking between the MHC-I and the modeled epitope, (c) energy minimization, (d) separation of MHC-I and modeled epitope and (e) second molecular docking as a final refinement.

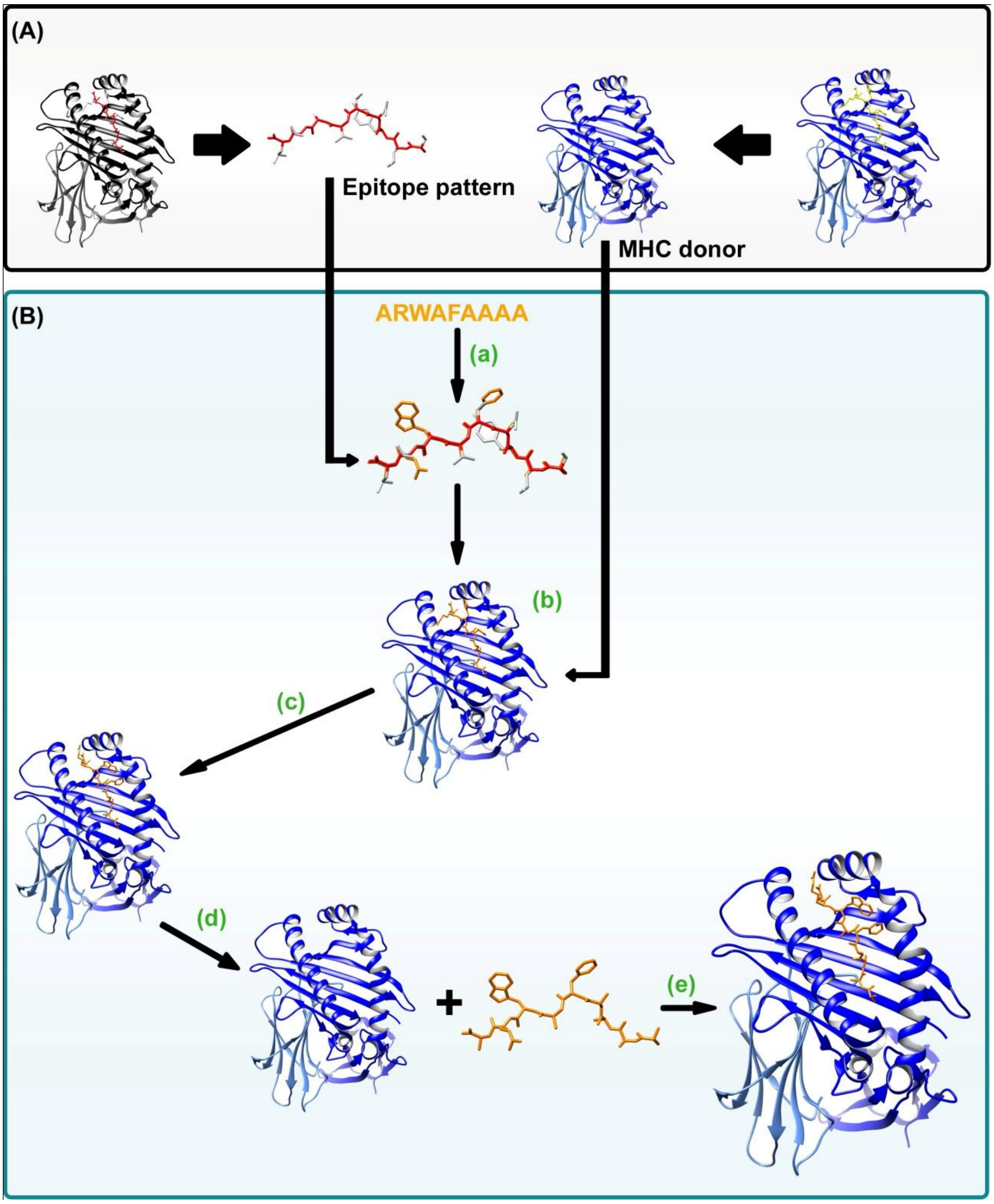

Supplementary Table S1. List of the 135 structures from PDB**.** Each structure was used to reproduce and validate DockTope, based on Cα and all atoms RMSD values for the epitope.

MHC-I Structure	PDB ID	Amino Acid Sequence	Sequence Length	RMSD (Cα)	RMSD (All atoms)
HLA-A*02:01	1B0G	ALWGFFPVL	9	1.349	2.446
HLA-A*02:01	1DUZ	LLFGYPVYV	9	0.996	2.739
HLA-A*02:01	1EEY	ILSALVGIV	9	0.557	1.053
HLA-A*02:01	1EEZ	ILSALVGIL	9	0.61	1.273
HLA-A*02:01	1HHG	TLTSCNTSV	9	1.082	2.391
HLA-A*02:01	1I1F	FLKEPVHGV	9	0.681	1.607
HLA-A*02:01	1I1Y	YLKEPVHGV	9	0.564	1.589
HLA-A*02:01	1I7R	FAPGFFPYL	9	1.532	3.575
HLA-A*02:01	1I7T	ALWGVFPVL	9	1.458	2.485
HLA-A*02:01	1I7U	ALWGFVPVL	9	0.836	1.566
HLA-A*02:01	1QEW	FLWGPRALV	9	0.907	2.038
HLA-A*02:01	1QR1	IISAVVGIL	9	1.39	2.053
HLA-A*02:01	1S8D	SLANTVATL	9	0.355	1.239
HLA-A*02:01	1S9X	SLLMWITQA	9	0.856	1.713
HLA-A*02:01	1S9y	SLLMWITQS	9	0.88	1.819
HLA-A*02:01	1T1W	SLFNTIAVL	9	0.327	1.409
HLA-A*02:01	1T1X	SLYLTVATL	9	0.417	1.239
HLA-A*02:01	1T1Y	SLYNVVATL	9	0.445	1.275
HLA-A*02:01	1T1Z	ALYNTAAAL	9	0.649	1.378
HLA-A*02:01	1T20	SLYNTIATL	9	0.453	1.395
HLA-A*02:01	1TVB	ITDQVPFSV	9	1.217	1.794
HLA-A*02:01	1TVH	IMDQVPFSV	9	1.093	1.813

HLA-A*02:02	2GIT	LLFGKPVYV	9	1.039	1.732
HLA-A*02:01	2GTW	LAGIGILTV	9	3.129	3.419
HLA-A*02:01	2GTZ	ALGIGILTV	9	1.435	1.493
HLA-A*02:01	2V2W	SLYNTVATL	9	0.605	1.253
HLA-A*02:01	2V2X	SLFNTVATL	9	1.384	2.403
HLA-A*02:01	2VLL	GILGFVFTL	9	0.788	2.475
HLA-A*02:01	2X4O	KLTPLCVTL	9	0.965	1.684
HLA-A*02:01	2X4S	AMDSNTLEL	9	1.258	2.042
HLA-A*02:01	2X4U	ILKEPVHGV	9	0.409	1.170
HLA-A*02:01	3D25	VLHDDLLEA	9	0.559	1.489
HLA-A*02:01	3FQT	GLLGSPVRA	9	1.135	1.652
HLA-A*02:01	3GSO	NLVPMVATV	9	0.603	1.337
HLA-A*02:01	3GSQ	NLVPSVATV	9	0.522	0.959
HLA-A*02:01	3GSR	NLVPVVATV	9	0.535	1.033
HLA-A*02:01	3GSU	NLVPTVATV	9	0.702	1.067
HLA-A*02:01	3GSV	NLVPQVATV	9	0.439	0.996
HLA-A*02:01	3GSW	NLVPMVAAV	9	0.883	1.694
HLA-A*02:01	3GSX	NLVPMVAVV	9	0.587	1.38
HLA-A*02:01	3H7B	MLWGYLQYV	9	0.684	2.449
HLA-A*02:01	3HPJ	RMFPNAPYL	9	1.111	1.492
HLA-A*02:01	3I6G	GLMWLSYFV	9	0.463	3.124
HLA-A*02:01	3KLA	SLLMWITQL	9	0.81	1.926
HLA-A*02:01	3MR9	NLVPAVATV	9	0.655	0.995
HLA-A*02:01	3MRB	NLVPMVHTV	9	0.681	1.497
HLA-A*02:01	3MRC	NLVPMCATV	9	1.63	2.613
HLA-A*02:01	3MRD	NLVPMGATV	9	1.608	2.537
HLA-A*02:01	3MRE	GLCPLVAML	9	1.093	2.092
HLA-A*02:01	3MRG	CINGVCWTV	9	0.978	1.623

HLA-A*02:01	3MRH	CISGVCWTV	9	0.853	2.576
HLA-A*02:01	3MRI	CINMWCWTV	9	1.481	3.778
HLA-A*02:01	3MRJ	CINGMCWTV	9	0.667	1.106
HLA-A*02:01	3MRK	PLFQVPEPV	9	0.696	1.299
HLA-A*02:01	3MRL	CINGVVWTV	9	0.606	1.9
HLA-A*02:01	3MYJ	YMFPNAPYL	9	1.659	1.858
HLA-A*02:01	3PWJ	LLYGFVNYV	9	0.804	2.492
HLA-A*02:01	3PWL	LGYGFVNYI	9	0.902	2.954
HLA-A*02:01	3PWN	LLYGFVNYI	9	0.733	2.355
HLA-A*02:01	3QFD	AAGIGILTV	9	1.377	1.421
HLA-A*02:01	3REW	CLGGLLTMV	9	1.158	2.224
HLA-A*02:01	3TO2	LACFVLAAV	9	1.006	2.79
HLA-A*02:01	3V5D	KVAELVHFL	9	1.099	2.378
HLA-A*02:01	3V5H	KVAEIVHFL	9	0.592	1.656
HLA-A*02:01	3V5K	KVAELVWFL	9	0.837	1.973
HLA-A*02:01	4I4W	ILAKFLHRL	9	1.219	3.494
HLA-A*02:01	4K7F	VCWGELMNL	9	0.971	1.713
HLA-A*02:01	4L3C	YLLMWITQV	9	1.003	2.273
HLA-B*27:05	1HSA	ARAAAAAAA	9	0.622	0.635
HLA-B*27:05	1JGE	GRFAAAIAK	9	0.557	0.894
HLA-B*27:05	1OGT	RRKWRRWHL	9	0.928	2.518
HLA-B*27:05	1UXS	RRRWRRLTV	9	1.685	4.355
HLA-B*27:05	1W0V	RRLPIFSRL	9	0.754	2.163
HLA-B*27:05	2A83	RRRWHRWRL	9	1.533	4.259
HLA-B*27:05	2BSR	RRIYDLIEL	9	0.673	1.816
HLA-B*27:05	2BST	SRYWAIRTR	9	0.686	2.637
HLA-B*27:05	3BP4	IRAAPPPLF	9	2.061	2.710
HLA-B*27:05	3LV3	SRRWRRWNR	9	0.774	2.994

H2-Db (9-mer epitope)	1BZ9	FAPGVFPYM	9	1.693	2.757
H2-Db (9-mer epitope)	1CE6	FAPGNYPAL	9	1.225	1.822
H2-Db (9-mer epitope)	1FFO	AAVYNFATM	9	0.852	1.694
H2-Db (9-mer epitope)	1FFP	SAVYNFATM	9	1.009	1.462
H2-Db (9-mer epitope)	1FG2	KAVYNFATC	9	0.379	2.034
H2-Db (9-mer epitope)	1HOC	ASNENMETM	9	0.744	1.658
H2-Db (9-mer epitope)	1INQ	SSVVGVWYL	9	1.18	2.6
H2-Db (9-mer epitope)	1JPG	FQPQNGQFI	9	0.5	1.109
H2-Db (9-mer epitope)	1JUF	SSVIGVWYL	9	1.182	2.647
H2-Db (9-mer epitope)	1S7U	KAVYNFATM	9	0.331	2.089
H2-Db (9-mer epitope)	1S7V	KAVYNLATM	9	1.135	2.339
H2-Db (9-mer epitope)	1S7W	KALYNFATM	9	0.271	2.064
H2-Db (9-mer epitope)	1S7X	KAVFNFATM	9	0.371	1.727
H2-Db (9-mer epitope)	1ZHB	KALYNYAPI	9	0.291	2.09
H2-Db (9-mer epitope)	2ZOK	ASLWNGPHL	9	0.592	1.226
H2-Db (9-mer epitope)	3CC5	KVPRNQDWL	9	0.308	1.856
H2-Db (9-mer epitope)	3CCH	EGSRNQDWL	9	0.495	2.288
H2-Db (9-mer epitope)	3CH1	EGPRNQDWL	9	0.656	2.214
H2-Db (9-mer epitope)	3CPL	ASNENAETM	9	0.845	1.769
H2-Db (9-mer epitope)	3FTG	ASAENMETM	9	0.912	1.518
H2-Db (9-mer epitope)	3QUK	KAVANFATM	9	0.454	1.742
H2-Db (9-mer epitope)	3QUL	KAVSNFATM	9	0.377	1.489
H2-Db (9-mer epitope)	3TBS	KAPANFATM	9	0.47	1.893
H2-Db (9-mer epitope)	3TBT	KAPSNFATM	9	0.336	1.536
H2-Db (9-mer epitope)	3TBV	KGPANFATM	9	0.653	1.912
H2-Db (9-mer epitope)	3TBW	KGPSNFATM	9	0.781	1.890
H2-Db (9-mer epitope)	3TBY	KAPFNFATM	9	0.436	2.288
H2-Db (9-mer epitope)	4HUU	ASNENIETM	9	0.695	2.005

H2-Db (9-mer epitope)	4HUV	ASNENWETM	9	0.64	1.792
H2-Db (9-mer epitope)	4HUW	ASNENTETM	9	0.537	1.72
H2-Db (9-mer epitope)	4L8C	ASDENMETM	9	0.639	1.517
H2-Db (9-mer epitope)	4L8D	ASNEDMETM	9	0.537	1.506
H2-Db (9-mer epitope)	4NSK	KAPYNFATM	9	0.629	2.416
H2-Db (10-mer epitope)	1N3N	SALQNAASIA	10	0.855	1.395
H2-Db (10-mer epitope)	1WBX	SQLKNNAKEI	10	0.441	1.685
H2-Db (10-mer epitope)	1YN6	SSLENFRAYV	10	0.315	2.54
H2-Db (10-mer epitope)	1YN7	SSLENFAAYV	10	0.233	0.937
H2-Db (10-mer epitope)	3L3H	SSLENARAYV	10	0.355	1.822
H2-Kb	1G7Q	SAPDTRPA	8	0.824	2.678
H2-Kb	1KJ3	KVITFIDL	8	1.322	2.298
H2-Kb	1LEG	EQYKFYSV	8	0.836	2.494
H2-Kb	1LK2	GNYSFYAL	8	0.653	1.778
H2-Kb	1RK0	SSIEFARL	8	0.692	1.601
H2-Kb	1RK1	SEIEFARL	8	0.658	1.504
H2-Kb	1VAC	SIINFEKL	8	0.416	1.455
H2-Kb	2CLV	SQYYYNSL	8	1.292	2.128
H2-Kb	2CLZ	INFDFNTI	8	1.442	1.849
H2-Kb	2FO4	SAPDFRPL	8	0.787	2.494
H2-Kb	3FOL	VNDIFERI	8	1.521	2.35
H2-Kb	3FON	VNDIFEAI	8	1.216	2.153
H2-Kb	3TID	AVYNFATM	8	1.367	2.135
H2-Kb	3TIE	AIVNYANL	8	1.25	1.438
H2-Kb	3P4M	YTVKYPNL	8	1.544	2.563
H2-Kb	3P4N	YTVKFPNM	8	1.604	2.172
H2-Kb	3P4O	YTAKYPNL	8	1.431	2.415
H2-Kb	3P9M	SIIGFEKL	8	1.341	1.559

H2-Kb	3PAB	EIINFEKL	8	1.312	2.392
**Mean ± SD**				**0.882 Å ± 0.437 Å**	**1.964 Å ± 0.655 Å**
